# Suprachiasmatic Nucleus Interaction with the Arcuate Nucleus; Essential for Organizing Physiological Rhythms

**DOI:** 10.1523/ENEURO.0028-17.2017

**Published:** 2017-03-24

**Authors:** Frederik N. Buijs, Mara Guzmán-Ruiz, Luis León-Mercado, Mari Carmen Basualdo, Carolina Escobar, Andries Kalsbeek, Ruud M. Buijs

**Affiliations:** 1 Instituto de Investigaciones Biomedicas, UNAM, Ciudad Universitaria, 04510 Mexico DF, Mexico; 2 Departamento de Anatomia, Facultad de Medicina, UNAM, Ciudad Universitaria, 04510 Mexico DF, Mexico; 3 Netherlands Institute for Neuroscience, an Institute of the Royal Netherlands Academy of Arts and Sciences, Amsterdam 1105 BA, The Netherlands

**Keywords:** circadian rhythm, clock genes, melatonin, network, oscillator coupling, temperature

## Abstract

The suprachiasmatic nucleus (SCN) is generally considered the master clock, independently driving all circadian rhythms. We recently demonstrated the SCN receives metabolic and cardiovascular feedback adeptly altering its neuronal activity. In the present study, we show that microcuts effectively removing SCN-arcuate nucleus (ARC) interconnectivity in Wistar rats result in a loss of rhythmicity in locomotor activity, corticosterone levels, and body temperature in constant dark (DD) conditions. Elimination of these reciprocal connections did not affect SCN clock gene rhythmicity but did cause the ARC to desynchronize. Moreover, unilateral SCN lesions with contralateral retrochiasmatic microcuts resulted in identical arrhythmicity, proving that for the expression of physiological rhythms this reciprocal SCN-ARC interaction is essential. The unaltered SCN c-Fos expression following glucose administration in disconnected animals as compared to a significant decrease in controls demonstrates the importance of the ARC as metabolic modulator of SCN neuronal activity. Together, these results indicate that the SCN is more than an autonomous clock, and forms an essential component of a larger network controlling homeostasis. The present novel findings illustrate how an imbalance between SCN and ARC communication through circadian disruption could be involved in the etiology of metabolic disorders.

## Significance Statement

The suprachiasmatic nucleus (SCN) is generally considered the master clock, independently coordinating all circadian rhythms. The present study challenges that view and shows that for a proper functioning circadian system, SCN interaction with the arcuate nucleus (ARC) is essential. We observed that interruption of SCN-ARC communication does not affect SCN rhythmicity but causes the ARC to desynchronize. This desynchrony between SCN and ARC results in animals losing activity, temperature, and corticosterone rhythmicity. As the ARC is an essential metabolic integration center, this might explain how chronic circadian or metabolic disruptions through ill-timed eating habits or shift work could cause desynchrony among hypothalamic oscillators and result in disease.

## Introduction

The suprachiasmatic nucleus (SCN) receives light input through direct synaptic connections from the retina; these photic cues attune the SCN rhythm with the environment. The SCN neuronal network consists of ∼20,000 tightly coupled neurons (Webb et al., 2009), with most neurons expressing their own endogenous rhythm through phasic clock gene expression (Reppert and Weaver, 2002). It is proposed that through multi phasic relationships between neuronal oscillators within the SCN, different physiologic rhythms can be timed and entrained to follow circadian patterns (Quintero et al., 2003; Kriegsfeld et al., 2004). In view of existing interactions between different hypothalamic nuclei and the SCN, one could presume the SCN as element of the hypothalamus in a similar fashion, i.e., the SCN not only leading but taking part in a hypothalamic network of coupled oscillators generating circadian rhythmicity. Indeed, the SCN receives a myriad of nonphotic input, arousal (Antle and Mistlberger, 2000), feeding behavior (Abe et al., 1989; Mendoza, 2007), locomotor activity (Edgar et al., 1991; Marchant and Mistlberger, 1995), immune function (O'Callaghan et al., 2012; Guerrero-Vargas et al., 2014), blood pressure (Peters et al., 1994; Buijs et al., 2014), and melatonin (Armstrong, 1989; Pitrosky et al., 1999), which are all able to adjust and synchronize the SCN. The SCN is capable of receiving this feedback through its large array of reciprocal neuronal connections with, e.g., the arcuate nucleus (ARC; Saeb-Parsy et al., 2000; Yi et al., 2006), intergeniculate leaflet (IGL; Janik and Mrosovsky, 1994; Saderi et al., 2012), nucleus tractus solitarius (Buijs et al., 2014), dorsal raphe (Shioiri et al., 1991), and dorsomedial hypothalamus (DMH; Acosta-Galvan et al., 2011), allowing these nuclei to convey nonphotic feedback to the SCN and thus adjusting circadian rhythmicity. Therefore, it is enticing to propose that the SCN is part of or central in a larger, complex neuronal network regulating circadian physiology and behavior.

We thus hypothesized that generating and synchronizing physiologic circadian rhythms depends on the integration of photic and nonphotic feedback to the SCN through existing strong neuronal interconnectivity with other hypothalamic nuclei. In view of recent observations that lesions targeted at specific neuronal populations in the ARC resulted in deteriorated temperature, feeding, and sleep rhythms (Li et al., 2012; Wiater et al., 2013), and the observations that the SCN induces a rhythm in the ARC (Guzmán-Ruiz et al., 2014; Herrera-Moro et al., 2016), and the ARC influences the activity of the SCN (Yi et al., 2006), we hypothesized that ARC-SCN reciprocity is an essential feedback pathway for the SCN, adjusting its output and facilitating changes in its neural activity in response to physiologic and behavioral activity. To test this hypothesis, we made retrochiasmatic knife cuts to isolate the ARC from the SCN, preventing their reciprocal communication; we measured specific physiologic output, along with central and peripheral clock gene expression. Under constant dark (DD) conditions such an SCN-ARC dissection resulted in animals showing complete arrhythmicity in behavior, temperature, and corticosterone secretion despite a rhythmic SCN and persistent rhythmic melatonin secretion. Unilateral SCN lesions combined with contralateral knife cuts provided identical outcomes. Immunohistochemistry and neuronal tracing confirmed successful disconnection of the SCN-ARC interaction. In addition, we demonstrated the IGL-SCN projections and SCN-subparaventricular zone (SPZ), paraventricular nucleus (PVN) and dorsomedial hypothalamic (DMH) projections to be unaffected by the knife cuts. In conclusion, the present results demonstrate the interaction between SCN and ARC to be crucial for the expression of select circadian rhythms and that the circadian network extends beyond the boundaries of the SCN.

## Materials and Methods

### Animals and ethical approval

Experiments were performed on male Wistar rats (∼250g) housed individually on a 12/12 h light/dark (LD) cycle (lights on, 7 A.M.) in a controlled environment. Rats were given food and water *ad libitum* unless otherwise stated. Piezoelectric motion sensors underneath the cages monitored locomotor activity and temperature data were acquired using ibuttons (Maxim) surgically inserted into the abdominal cavity.

### Surgery

All animals undergoing surgery were anesthetized with ketamine (50 mg/kg) and xylazine (2 mg/kg) (Pisa-Agropecuaria S.A. de C.V.). Initial preliminary experiments were performed using bilateral thermic ARC lesions using two Teflon coated electrodes 0.2 mm in diameter with excoriated tip. Animals were placed in a stereotactic frame (toothbar, −4 mm; arm, 6°; coordinates: −2.0 from bregma; ±1.2 lateral from midline; 8.2–8.6 mm below brain surface) and an electric current of 0.3 mA was passed for 60 s, sufficient to eliminate the ARC bilaterally.

**Figure 1.**
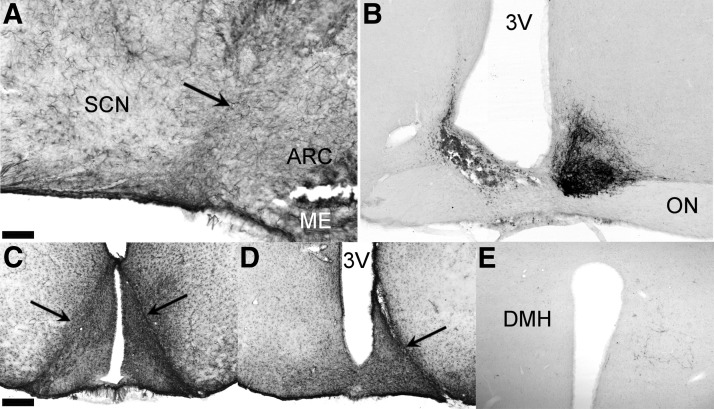
Representative sagittal and coronal sections of the hypothalamus illustrating the 45° angle knife cut and its effect established through GFAP and VIP staining. ***A***, Sagittal GFAP-stained section just lateral to the third ventricle with minimal glial damage around the site of incision indicated by a black arrow. ***B***, Unilateral SCN lesion in combination with a contralateral knife cut with VIP staining showing the contralateral SCN intact. ***C***, Shows GFAP staining of the most caudal reach of the knife isolating the ARC from the SCN. ***D***, Unilateral RC-cut isolating the ARC contralateral to the SCN lesion shown in *B*. ***E***, Unilateral VIP innervation of the DMH on the side of the RC-cut demonstrating effective unilateral innervation as compared with the loss of innervation on the SCN-lesioned side (left). Scale bar, 100 μm (***A***), 90 μm (***B***), 250 μm (***C*** and ***D***), and 130 μm (***E***). ME, median eminence; 3V, third ventricle.

Medial, retrochiasmatic knife cuts (RC cut) were made using a small knife derived from two telescoped needles with a 45° angle, sharpened tip. RC cuts were intended to disrupt fibers of passage, without causing extensive damage to the neuronal populations in the retrochiasmatic area. Animals were placed in a stereotactic frame (toothbar, −4 mm; knife placed perpendicular to midline, −1.1 mm from bregma), the knife was lowered ventrally until the sphenoid bone was reached (∼8.2 mm below brain surface) and turned 90° caudally, in duplex (Fig. 1*A*,*C*). In SHAM animals, the surgical procedure was identical but the knife lowered without gyrating.

To further investigate the phenotype resulting from the disrupted interaction between SCN and ARC, unilateral SCN lesions were made, jointly with a contralateral 90° knife cut (SCN_X_ARC^Cut^; Fig. 1*B*,*D*). Animals were subjected to the same experimental protocols as animals receiving retrochiasmatic knife cuts (RC cut). Finally, we performed a glial fibrillary acidic protein (GFAP) immunostaining to show the extent of glial scarring, thus assessing the magnitude of damage caused by placed knife cuts. This demonstrated only minor glial damage to adjacent areas in RC-cut-operated animals (Fig. 1*A*,*C*,*D*).

In testing to what degree severing SCN-ARC efferents eliminate neuronal innervation of the SCN, we performed unilateral cholera toxin B (CtB), ARC injections in animals with and without ipsilateral retrochiasmatic knife cut. CtB injections (Invitrogen) were made using the above coordinates for the ARC. With a glass micropipette (∼30-μm tip), 0.05μl 1% CtB was pressure injected (10 mbar, 5 s) and the micropipette left in place for 5 min to minimize tracer leakage.

#### Perioperative protocol

Before surgery, a 7 d LD/7 d DD baseline was acquired, and unless stated otherwise, all animals were kept under a 12 h [6 A.M. lights on (300 lux), 6 P.M. lights off] LD cycle. After surgery, a similar protocol was adhered to. Experimental (both RC cut and SCN_X_ARC^Cut^ animals) and SHAM animals were allowed to recover and kept in L/D for 8-10 d, followed by 7 d of DD, during which activity and temperature recordings were made. Of all animals operated (*n* = 49; RC-cut, SHAM and SCNxARCx animals) a total of 40 were included for further analysis. Only animals showing arrhythmic locomotor activity in DD through χ^2^ analysis and in which subsequent anatomic analysis demonstrated the knife cut to be placed medially, in the retrochiasmatic area, were included. In experiments performed during LD conditions, time is indicated as zeitgeber time (ZT), as the animals are entrained to light (zeitgeber). Experiments performed in DD conditions, time is indicated as circadian time (CT), indicating the animals endogenous rhythm of ∼24 h (circa dies) not entrained to external cues.

### Blood sample collection

Blood was drawn (200 μl) from the tail at ZT and CT 0, 6, 12, and 18; centrifuged for 5 min at 5000 rpm, and supernatant was frozen at 20°C, from which melatonin (IBL International) and corticosterone were assayed using an ^125^I RIA kit (MP Biomedicals). Rats were placed in a towel during blood withdrawal to minimize handling stress and they were habituated daily for 5 d before blood withdrawal; care was taken to draw blood within 30 s after taking the animal out of its home cage. Blood was sampled at CT/ZT 0 and 12 on the same day. At least 2 d later, blood was sampled at 6 and 18.

### Physiologic rhythmicity following SCN-ARC axis disruption

The different groups of animals were treated as follows. Preliminary pilot experiments were performed using bilateral thermic ARC lesions (*n* = 3). Thereafter, RC-cut (*n* = 16) and SHAM-operated animals (*n* = 18) were exposed to above-mentioned LD and DD conditions, during which locomotor activity and temperature were registered (*n* = 7 for both RC-cut and SHAM animals). The remaining animals (*n* = 9 for RC-cut and *n* = 11 for SHAM) were used for immunohistochemistry and *in situ* analysis. Following this initial registration period and to evaluate melatonin and corticosterone levels, two hormones reflecting direct influence of the SCN, blood samples were taken in LD at ZT0, ZT6, ZT12, and ZT18 (*n* = 5-7/time point). Blood was drawn from the tail 2 d apart at two different time points per animal to minimize stress. One week later, blood was drawn from each animal for DD blood serum analysis following 36 h of DD at CT0, CT6, CT12, and CT18 (*n* = 3-5/time point). Then animals were killed using an overdose of sodium pentobarbital (Sedal-Vet 65 mg/ml) livers were extracted, immediately put on dry ice and kept frozen at -80°C until further analysis, then animals were perfused transcardially with saline, followed by a freshly prepared 4% paraformaldehyde solution. Following perfusion fixation, brains were removed and preserved for histologic analysis and *in situ* hybridization (ISH), as stated below.

SCN_X_ARC^Cut^ animals (*n* = 6) underwent a similar protocol and likewise blood samples were taken at ZT0, ZT6, ZT12, and ZT18 (*n* = 5-6/time point). Due to technical issues we could record a full temperature registry of only four animals. Animals were killed and their brains extracted for immunohistochemistry to verify the extent of the lesion and knife cut.

### Testing the ARC-SCN interaction

To investigate the relevance of the feedback from the ARC to the SCN, we assessed whether metabolic signaling to the ARC would differently affect SCN activity, depending on whether animals received feedback from the ARC or not, allowing us to demonstrate that the ARC directly alters SCN neuronal activity in response to metabolic signals. RC-cut-operated rats were fasted for 48 h in LD (12 h lights on/off) followed by 5 ml of 3% oral glucose (0.05-g glucose/kg) intake given in a separate water bottle at ZT2 and killed 2 h later (ZT4). In two other groups, SHAM-operated (*n* = 4-6) and RC-cut animals (*n* = 4-6), animals were 48 h fasted or given *ad libitum* conditions. Before fasting, only animals receiving glucose were entrained to drink 3% glucose water during the light period at random time points during 5 d. All animals were killed at ZT4. In brain sections of the differently treated animals, the neuronal activity of the SCN and ARC was examined using c-Fos as activity marker.

### Immunohistochemistry

Following killing, brains were removed, post fixed for 24 h, cryoprotected in 30% sucrose for 48-72 h, frozen, and cut in coronal sections of 35 μm at –20°C. Free floating sections were processed for c-Fos (rabbit polyclonal, 1:40,000; Calbiochem), α-MSH (sheep, 1:10,000; Millipore Bioscience Research Reagents), AgRP (goat, 1:4000; Millipore Bioscience Research Reagents), VIP (rabbit, 1:2000; Buijs et al., 1989), Per1 (rabbit 1:2000, Santa Cruz Biotechnology), and CtB (rabbit, 1:2000; Sigma-Aldrich) immunohistochemistry using a avidin-biotin-peroxidase procedure followed by 3,3’-diaminobenzidine (DAB) staining. SHAM and experimental tissues were always processed together to avoid confounding by slight differences in staining.

### ISH

The primers were synthesized by Sigma-Aldrich with forward ‘5 ACCCCCTGCTATGTGTCTCA ‘3 and reverse ‘5 TCACTGGAGCCTGAAAGTGC ‘3 for Per1 with a base length of 569 bp. The reverse primers were labeled in 5’ with T7 polymerase promoter. An antisense DIG-RNA label was obtained using 200-ng purified PCR fragment for Per1, 2 µL of DIG-RNA labeling mix (Roche), 2-µL T7 RNA and polymerase (Roche), 2-µL 10× concentrated transcription buffer (Roche), and 40-U RNaseOUT (Invitrogen), then supplemented with MilliQ diethyl pyrocarbonate (DEPC) water until 20 µL. Following incubation, the labeling efficiency of the DIG-labeling reaction was determined in a spot assay. The RNA dilutions were spotted on Nylon Membrane (Zeta probe, Bio-Rad), incubated with anti-DIG POD (Roche) and developed with DAB. The RNA was compared with labeled control RNA (Roche).

Following a short rinse in PBS, sections were fixed in 4% paraformaldehyde in PBS for 5 min and treated with H2O_2_ 3% in PBS-DEPC for 30 min. Sections were washed three times for 5 min in PBS and incubated in 0.5-µg/mL proteinase K in PBS with Triton X-100 0.1% for 10 min at room temperature followed by 10 min postfixation in 4% paraformaldehyde in PBS.

Following washing and incubation in hibridization mix, overnight sections were rinsed and incubated with blocking solution 1% (Roche) in buffer 1 for 30 min to block nonspecific protein binding. Sections were washed and the signal was amplified with TSA plus biotin kit (PerkinElmer) according to instructions. Finally, sections were incubated with Streptavidin HRP (The Jackson Laboratory) 1:500 in buffer 1 for 30 min. Following three washes in buffer 1, the reaction was revealed with DAB-Nickel, and sections were mounted with Entelan.

### RT-qPCR

Total RNA was extracted from the liver using TRIzol reagent (life technologies) according to the manufacturer’s protocol. The concentration and RNA quality were confirmed with agarose gel . Total RNA (2500 ng) from the liver was reverse transcribed to single strand cDNA using SuperScript III first-strand synthesis (Invitrogen) according to the manufacturer’s protocol. The cDNA samples were quantified in Nanodrop and diluted in RNase free water at 250 ng/µl and stored at −20°C. For absolute quantification, we generated a standard calibration curve using highly purified standards that had been carefully quantified as to the amount or copy number. A standard curve was derived from setting out the threshold cycle against the number of RNA copies. The corresponding curves and logarithmic regression formulas were generated to replace necessary data obtained experimentally. Amplification was performed as follows: 1 μl of the first strand cDNA sample was mixed with 5-μl SYBR select master mix (Applied biosystems), 2-μl milliQ water, and 1-μl primers mix 10mM (Sigma). A StepOne real-time PCR system (Applied biosystems) was used to amplify the genes from each liver sample in triplicate on a 48-well reaction plate using the following protocol: 10-min denaturation at 95°C, 40 cycles of 15 s denaturation at 95°C and 60 s annealing and extension at 60°C. The sequences of primers used (designed by Sigma’s OligoArchitect program): forward ‘5 ACATTCCTAACACAACCAA ‘3 and reverse ‘5 TGCTTGTCATCATCAGAG ‘3 for Per1; forward ‘5 CCTACTCTGATAGTTCGTCTA ‘3 and reverse ‘5 ATCCTTGGTCGTTGTCTA ‘3 for Bmal1; forward ‘5 GGAGACTATATTAGGCGTTA ‘3 and reverse ‘5 CCTTCTGGATACCTTCTG ‘3 for Cry1.

### Quantification

Pictures were taken by using an Axioplan microscope (Zeiss) equipped with a digital color camera (Olympus DP25, Olympus). The SCN and ARC were manually outlined; the Fos-positive nuclear profiles were automatically detected by means of size and staining threshold detection. The same parameters were used for all experimental groups using ImageJ software (NIH). For each rat, two to three sections were measured ∼90 μm apart; the mean number of c-Fos-positive nuclear profiles from both SCN per section was calculated. ARC Per1-immunoreactivity (Per1-ir) was quantified in the same manner. Demarcating the ARC neuroanatomical landmarks were used to draw a triangular form in accordance to the rat brain atlas (Paxinos and Watson), also determining the equivalent sections between animals. All c-Fos or Per-1-ir positive nuclei were counted in this area. For SCN Per1 *in situ* quantification, a manual outline of the SCN was made, the mean optic density for that area was determined and the background optic density just outside the SCN was subtracted. For each rat, three sections were taken ∼90 μm apart. Only the sections with the highest Per1 expression were analyzed and compared.

### Statistics

All data were normally distributed and are expressed in mean ± SEM. Statistical comparisons were performed using GraphPad Prism (GraphPad software). Data were analyzed using one-way ANOVA when appropriate. Analyzing SCN and ARC neuronal activity in different metabolic conditions a two-way ANOVA was used for factor group, i.e., SHAM versus RC cut (two levels) and factor metabolic condition, i.e., *ad libitum*, fasting, and glucose (three levels). This was followed by a Tukey’s multicomparison *post hoc* test; *p* < 0.05 was deemed significant. χ^2^d (χ^2^) locomotor activity analysis was performed using SPAD9 (a program linked to our activity monitoring system).

A repeated measures (RM)-ANOVA was used for temperature and clock gene analysis with the factor group, i.e., SHAM versus RC cut (two levels) and time as a factor of RM (23 levels for temperature and four levels for clock genes). For corticosterone and melatonin, the factor group, i.e., SHAM versus RC-cut versus SCNxARCx (three levels), was also set against time as a factor of RM (four levels). For the RM-ANOVA, *p* < 0.05 was considered statistically significant. If a significant effect was reached, linear harmonic regression analysis was performed to determine circadian rhythmicity. CircWave version 1.4 software (Oster et al., 2006) was used employing an *F* test to validate rhythmicity with an assumed period of 24 h and a threshold value of 0.05 for α. For data visualization purposes where a significant rhythm in expression was detected, data were fitted with a sine wave function using GraphPad software.

## Results

### SCN-ARC axis disruption causes arrhythmicity in DD conditions

**Figure 2.**
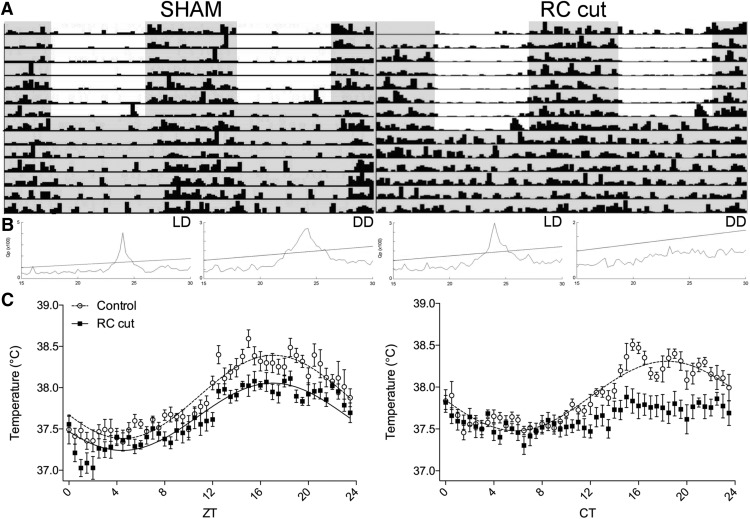
Arrhythmic locomotor activity and temperature observed in DD following retrochiasmatic knife cuts (RC cut). ***A***, Representative actograms of a SHAM-operated animal (left) and an RC-cut animal (right), in LD and DD conditions (shading indicates lights off). Following RC cuts, animals recovered in LD and showed normal diurnal rhythmicity, subsequent DD conditions rendered all RC-cut animals arrhythmic. ***B***, χ^2^ periodogram analysis of LD and DD periods of SHAM (left) and RC cut (right) animals. The slanted line in the periodogram indicates *p* = 0.01. ***C***, For RC-cut animals temperature was arrhythmic in DD. Note the strong temperature decrease in RC-cut animals between ZT0 and ZT2 in reaction to light during LD. The graph is a double plot of temperature data in SHAM and RC-cut animals in LD (left) and DD (right). Each value represents seven animals (mean ± SEM). Only where a significant rhythm in expression was detected, data were fitted with a sine wave function (*p* < 0.05, *F* test).

Animals kept in DD following bilateral ARC lesions demonstrated a clear loss of rhythm in locomotor activity (data not shown). Since these animals maintained a perfect rhythm under LD conditions we hypothesized that in the absence of light, the SCN requires an additional synchronizing stimulus from the ARC to drive its output. To investigate this hypothesis, coronal retrochiasmatic knife cuts (RC cuts) were made, leaving both ARC and SCN intact but severing their direct neuronal connections. χ^2^d periodogram analysis of locomotor activity in light/dark (LD 12 h lights on/off, 7 d, *n* = 7), showed a significant diurnal rhythm in both SHAM and RC-cut animals; however, in DD (7 d, *n* = 7), all RC-cut animals were rendered arrhythmic (Fig. 2; Table 1). When RC cuts just missed the medial, most ventral part of the retrochiasmatic area, various phenotypes of rhythmicity could be observed during DD. Only animals showing arrhythmic locomotor activity in DD and in which subsequent anatomic analysis demonstrated the knife cut to be placed medially, in the retrochiasmatic area, were included for further analysis.

**Table 1.** Analysis of activity and temperature rhythms in SHAM, RC-cut, and SCN_X_ARC^Cut^ animals during LD and DD conditions

		LD			DD		
Control	RC cut	SCN_X_ARC^Cut^	Control	RC cut	SCN_X_ARC^Cut^		
Activity	Acrophase (ZT/CT)	17.23 ± 0.4	16.6 ± 0.55	15.93 ± 0.38	17.88 ± 0.63	—	—
	Amplitude	1.79 ± 0.15	1.68 ± 0.22	1.42 ± 0.12	1.36 ± 0.07#	—	—
	R^2^	0.5	0.45	0.5	0.42	0.05	0.12
Rhythmic	7/7	7/7	6/6	7/7	0/7	0/6
Temperature	Mean (°C)	37.43 ± 0.04	37.66 ± 0.06*	37.77 ± 0.05**	37.43 ± 0.04	37.63 ± 0.0.07	37.79 ± 0.02**
	Acrophase (ZT/CT)	16.9 ± 0.36	16.95 ± 0.56	16.06 ± 0.42	17.99 ± 0.29	—	—
	Amplitude	0.49 ± 0.032	0.38 ± 0.04	0.33 ± 0.03	0.42 ± 0.04	—	—
	R^2^	0.67	0.59	0.54	0.59	0.14	0.23
Rhythmic	7/7	7/7	4/4	7/7	1/7	0/4

**Figure 3.**
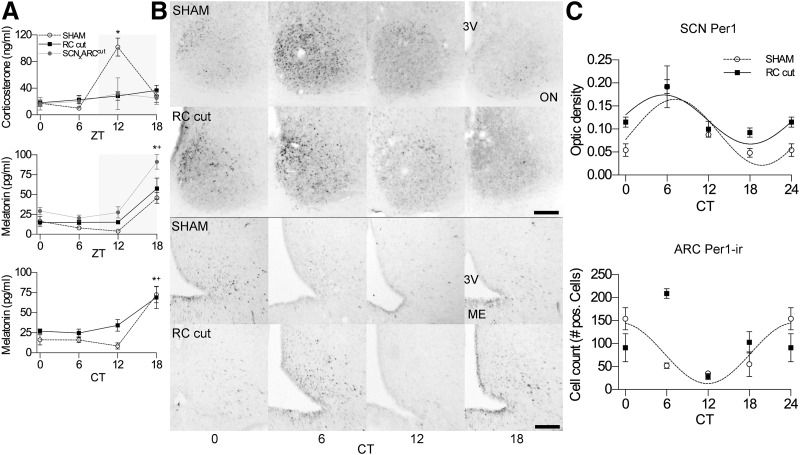
In RC-cut animals corticosterone rhythm was lost while melatonin remained intact similar to SCN rhythmicity. Rhythm of ARC *Per1* expression was attenuated, demonstrating SCN-ARC desynchronization. ***A***, Corticosterone levels showed a significant peak at ZT12 in SHAM but not RC-cut or SCN_X_ARC^Cut^ animals, indicating loss of rhythmicity. Melatonin maintained its rhythm in LD and DD in both SHAM and experimental animals. Data represents *N* = 5-7, and *N* = 4-6 for the SCN_X_ARC^Cut^ group (mean ± SEM). *p < 0.05 (SHAM), +*p* < 0.05 (Experimental), ANOVA analysis. ***B***, Representative photomicrographs of SCN Per1 mRNA expression (above) and ARC Per1-ir (below) in SHAM and RC-cut animals. Scale bar, 90 μm. 3V, third ventricle; ON, optic nerve; ME, median eminence. ***C***, *Per1* mRNA analysis of the SCN demonstrates significant circadian rhythmicity in RC-cut (*p* < 0.05) and in SHAM animals (*p* < 0.01). The ARC failed to show a significant rhythm in RC-cut animals (*p* > 0.05) as compared with SHAM. The higher point at CT6 suggests a phase advance; however, an *F* test failed to show a significant circadian rhythm. Data represents *N* = 3-4 with a double-plot of CT0 at CT24. Only where a significant rhythm in expression was detected, data were fitted with a sine wave function (*p* < 0.05, *F* test).

Temperature analysis showed a robust diurnal rhythm in LD in SHAM-operated (RM-ANOVA, *F*_(47,288)_ = 8.01909, *p* < 0.0001; *F* test, *p* < 0.0001) and RC-cut animals (*F*_(47,288)_ = 4.7408, *p* < 0.0001; *F* test, *p* < 0.0001). However, in DD RC-cut animals, failed to demonstrate a significant difference between time points (*F*_(47,288)_ = 1.195, *p* = 0.1925) as compared with SHAM animals (*F*_(47,288)_ = 4.492, *p* < 0.0001; *F* test, *p* < 0.0001; Fig. 2*C*; Table 1). Thus, the pronounced rhythm seen during LD conditions, in contrast to the absent rhythms in DD, raises the question whether all functional SCN output was disrupted in DD and rescued in LD. Therefore, two to three weeks after surgery, corticosterone and melatonin levels were analyzed from tail blood samples at ZT0, ZT6, ZT12, and ZT18. Surprisingly, despite their rhythmic temperature and locomotor activity in LD, animals with an SCN-ARC RC cut showed no significant variation among different time points in corticosterone levels during LD (RM-ANOVA, *F*_(3,22)_ = 1.707, *p* = 0.1947), contrary to SHAM (*F*_(3,21)_ = 25.45, *p* < 0.0001; Fig. 3*A*) with a peak at ZT12 (*p* < 0.0001). However, the same animals did express a significant effect of time in melatonin levels (*F*_(3,19)_ = 7.219, *p* = 0.002) similar to SHAM (*F*_(3,16)_ = 15.47, *p* < 0.0001; Fig. 3*A*) with a peak at ZT18 (*p* = 0.0025). Blood samples collected 36 h following DD at CT0, CT6, CT12, and CT18 demonstrated that melatonin, despite absent temperature and activity rhythms, continued to be rhythmic in DD in both SHAM (*F*_(3,13)_ = 21.04, *p* < 0.0001) and RC-cut animals (*F*_(3,12)_ = 4.665, *p* = 0.022; Fig. 3*A*) with a peak at CT18 (*p* = 0.0221). Considering that corticosterone was significantly arrhythmic in LD, duplicate measurements in DD were not regarded as additive. These observations demonstrate that, although the majority of the determined physiologic rhythms were absent in DD conditions, melatonin secretion occurs independent of the SCN-ARC interaction. Furthermore, the present results show that corticosterone rhythm, known to depend on the integrity of the SCN, also depends on the integrity of SCN-ARC communication.

### Interaction between the SCN and ARC is essential for rhythmicity

To confirm that only the interaction between the SCN and ARC was essential for rhythmicity and the observed arrhythmicity was not simply caused by damage to the retrochiasmatic area, we made unilateral electrolytic SCN lesions in combination with contralateral 90**°** retrochiasmatic knife cuts. Thus, maintaining the ARC, the unilateral SCN and unilateral retrochiasmatic area intact, while also the unilateral afferents and efferents of ARC and retrochiasmatic area were not disrupted, the reciprocal communication between SCN and ARC was still effectively removed (Fig. 1*B*,*D*). Contrary to unilateral SCN or mediobasal hypothalamus lesions, shown to be ineffective in changing rhythmicity (Gerkema et al., 1990), our unilateral SCN-lesion combined with contralateral ARC-isolation (SCN_X_ARC^Cut^) animals were behaviorally arrhythmic in DD as was temperature (Table 1). As with RC-cut animals, SCN_X_ARC^Cut^ animals showed arrhythmic corticosterone (*F*_(3,17)_ = 1.048, *p* = 0.3967) but rhythmic melatonin levels in LD (*F*_(3,11)_ = 26.38, *p* < 0.0001; Fig. 3*A*) according to ANOVA analysis (see details on statistical analysis table 2). This confirmed that the observed arrhythmicity in RC-cut animals was indeed due to elimination of SCN-ARC intercommunication.

**Table 2.** Statistical table

Figure	Panel	Distribution	Test	*p* value
2	Ca	Normal distribution	RM-ANOVA *F* test	*F*_(47,288)_ = 8.01909, *p* < 0.0001 *p* < 0.0001
	Ca	Normal distribution	RM-ANOVA *F* test	*F*_(47,288)_ = 4.7408, *p* < 0.0001 *p* < 0.0001
	Cb	Normal distribution	RM-ANOVA *F* test	*F*_(47,288)_ = 4.492, *p* < 0.0001 *p* < 0.0001
	Cb	Normal distribution	RM-ANOVA	*F*_(47,288)_ = 1.195, *p* = 0.1925
3	Aa	Normal distribution	RM-ANOVA	*F*_(3,21)_ = 25.45, *p* < 0.0001
	Aa	Normal distribution	RM-ANOVA	*F*_(3,22)_ = 1.707, *p* = 0.1947
	Aa	Normal distribution	RM-ANOVA	*F*_(3,17)_ = 1.048, *p* = 0.3967
	Ab	Normal distribution	RM-ANOVA	*F*_(3,16)_ = 15.47, *p* < 0.0001
	Ab	Normal distribution	RM-ANOVA	*F*_(3,19)_ = 7.219, *p* = 0.002
	Ab	Normal distribution	RM-ANOVA	*F*_(3,11)_ = 26.38, *p* < 0.0001
	Ac	Normal distribution	RM-ANOVA	*F*_(3,13)_ = 21.04, *p* < 0.0001
	Ac	Normal distribution	RM-ANOVA	*F*_(3,12)_ = 4.665, *p* = 0.022
3	Ca	Normal distribution	RM-ANOVA *F* test	*F*_(3,16)_ = 0.9766, *p* = 0.4283 *F*_(3,16)_ = 15.23, *p* < 0.0001 *F*_(1,16)_ = 4.188, *p* = 0.0575 *p* = 0.0099/*p* = 0.0123
	Cb	Normal distribution	RM-ANOVA *F* test	*F*_(3,16)_ = 11.14, *p* = 0.0003 *F*_(3,16)_ = 11.80, *p* = 0.0003 *F*_(1,10)_ = 6.136, *p* = 0.0248 *p* = 0.01305/*p* = 0.2178
4	A	Normal distribution	RM-ANOVA *F* test	*F*_(3,8)_ = 4.43, *p* = 0.0410/*F*_(3,10)_ = 8.53, *p* = 0.0041 *p* = 0.0254/*p* = 0.0038
	B	Normal distribution	RM-ANOVA *F* test	*F*_(3,8)_ = 21.93, *p* = 0.0003/*F*_(3,10)_ = 2.56, *p* = 0.1134 *p* = 0.0043/*p* = 0.268
	C	Normal distribution	RM-ANOVA *F* test	*F*_(3,8)_ = 8.009, *p* = 0.0086/*F*_(3,10)_ = 2.373, *p* = 0.1316 *p* = 0.0023/*p* = 0.1372
5	Ca	Normal distribution	2ANOVA	*F*_(2,25)_ = 3.489, *p* = 0.0461 *F*_(2,25)_ = 9.341, *p* = 0.0009 *F*_(1,25)_ = 93.09, *p* < 0.0001
5	Cb	Normal distribution	2ANOVA	*F*_(2,25)_ = 15.59, *p* < 0.0001 *F*_(2,25)_ = 2.838, *p* = 0.0775 *F*_(1,25)_ = 3.6, *p* = 0.0694

RM-ANOVA, ANOVA (one-way or two-way); 2ANOVA, two-way ANOVA; *F* test, linear harmonic regression analysis.

### The SCN remains rhythmic following SCN-ARC deafferentation

Observing the circadian disruption of RC-cut animals in DD, we hypothesized that either (1) specific SCN output is altered, with the SCN depending on synchronized concomitant ARC output for adequate regulation of physiologic function; or (2) the robustness of SCN rhythmicity is affected, resulting in diminished clock function and circadian control, only revealed during DD conditions. We analyzed SCN *Per1* expression in DD conditions through ISH with animals killed 36 h after lights off at CT0, CT6, CT12, and CT18. RC-cut animals showed a slightly higher expression of *Per1* at all time points, but with equal amplitude of *Per1* expression as compared with SHAM animals (RM-ANOVA, *F*_(3,16)_ = 0.9766, *p* = 0.4283; Fig. 3*C*). Also, a preserved *Per1* circadian rhythmicity in both SHAM (*F*_(3,16)_ = 15.23, *p* < 0.0001; *F* test, *p* = 0.0099) and RC-cut animals (*F* test, *p* = 0.0123) was found. This demonstrates SCN rhythmicity is not significantly affected by SCN-ARC axis disruption.

**Figure 4.**
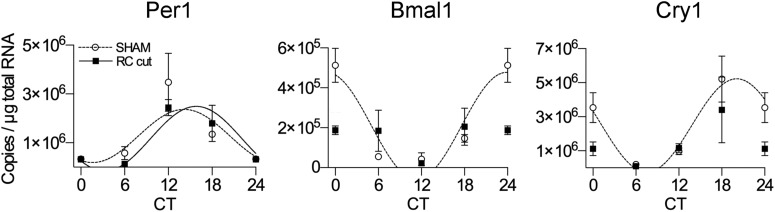
Peripheral liver clock genes in experimental animals show contrasting changes in rhythmicity as compared with SHAM animals. *Per1* gene expression in experimental animals showed no significant changes in rhythmicity (*p* < 0.01) as compared with SHAM (*p* < 0.05), contrary to *Bmal1* (*p* = 0.268) and *Cry1* (*p* = 0.137) failing to demonstrate rhythmicity as compared with SHAM animals (*p* < 0.05). Each value represents three to four animals (mean ± SEM). CT24 is a double-plot of CT0. Solid squares indicate RC-cut animals and open circles SHAM animals. Only where a significant rhythm in expression was detected, data were fitted with a sine wave function (*p* < 0.05, *F* test).

Next, we hypothesized that the rhythmic SCN, together with rhythmic melatonin levels, implies an intact autonomic control over peripheral clock gene rhythmicity, though to some extent other physiologic processes like corticosterone rhythm and food intake also have their impact on peripheral clock-gene rhythmicity. We measured clock gene expression in the liver of SHAM and experimental animals using RT-qPCR. *Per1* showed a clear circadian oscillation in both SHAM (RM-ANOVA, *F*_(3,8)_ = 4.43, *p* = 0.0410; *F* test, *p* = 0.0254) and RC-cut animals (*F*_(3,10)_ = 8.53, *p* = 0.0041; *F* test, *p* = 0.0038). However, *Bmal1* failed to show an effect of time (*F*_(3,10)_ = 2.56, *p* = 0.1134; *F* test, *p* = 0.268) in experimental animals as compared with SHAMs (*F*_(3,8)_ = 21.93, *p* = 0.0003; *F* test, *p* = 0.0043); likewise, Cry1 expression in experimental animals showed no effect of time, nor circadian rhythmicity (*F*_(3,10)_ = 2.373, *p* = 0.1316; *F* test, *p* = 0.1372) unlike in SHAMs (*F*_(3,8)_ = 8.009, *p* = 0.0086; *F* test, *p* = 0.0023; Fig. 4).

### The SCN and ARC desynchronize

Under normal conditions *Per1* expression in the ARC shows a diurnal rhythm *in vivo* (Shieh et al., 2005) with a peak around ZT18. To investigate whether the known rhythm of Per1 protein in the ARC would depend on neuronal interaction with the SCN we also investigated Per1 protein expression in the ARC after SCN deafferentation through RC cuts. This showed a significant difference between SHAM and RC-cut animals (RM-ANOVA, *F*_(1,10)_ = 6.136, *p* = 0.0248), a significant effect of time (*F*_(3,16)_ = 11.14, *p* = 0.0003) but also a significantly different time trend in ARC PER1 expression between SHAM en RC-cut animals (*F*_(3,16)_ = 11.80, *p* = 0.0003). Testing for circadian rhythmicity revealed that SHAM animals showed a clear rhythm (*F* test, *p* = 0.01305) with peak expression around CT23 (22.98 ± 0.73); however, in experimental animals PER1 failed to express circadian rhythmicity in the ARC (*F* test, *p* = 0.2178; Fig. 3*C*). Together with the preserved circadian *Per1* expression in the SCN, this indicates a significant desynchrony between SCN and ARC in RC-cut animals as compared with SHAM animals.

### RC cuts reveal altered metabolic SCN-ARC communication

**Figure 5.**
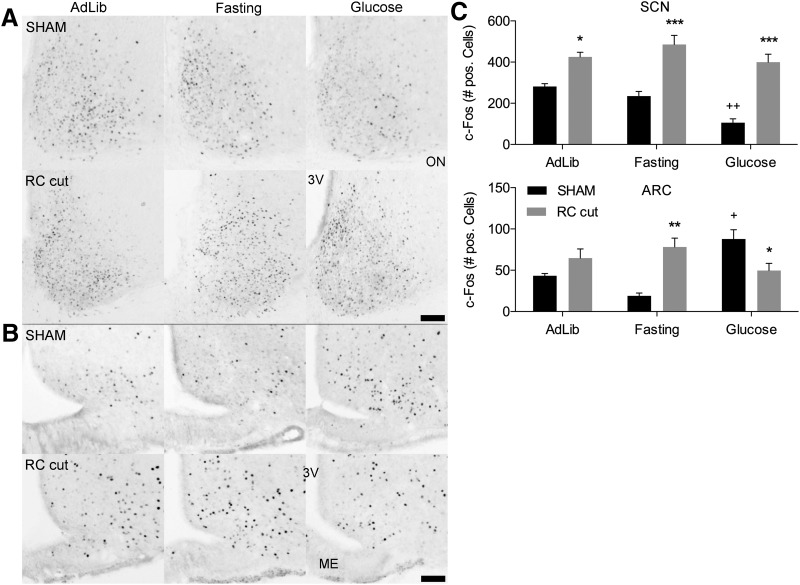
c-Fos staining of the SCN and ARC showing significant changes in Fos expression following glucose intake in SHAM animals, whereas experimental animals show no significant alterations in Fos expression. ***A***, Representative photomicrographs of SCN c-Fos expression in SHAM en RC-cut animals in *ad libitum*, fasting or fasting and glucose intake. ***B***, ARC c-Fos expression in SHAM en RC-cut animals during above stated conditions. ***C***, Bar charts of c-Fos quantification in the SCN and ARC of SHAM en RC-cut animals. All animals were killed at ZT4 following *ad libitum*, 48 h of fasting or fasting followed by 5-ml 3% glucose intake 2 h before killing. Each value in ***C*** represents three to four animals (mean ± SEM). Scale bar, 70 μm; *significant between group difference; +significant within group difference. */+*p* < 0.05, **/++*p* < 0.01, ***/+++*p* < 0.001 (ANOVA analysis).

To investigate whether ARC-SCN metabolic communication, was impaired by the RC cuts, we subjected animals to a protocol of fasting followed by a brief glucose stimulus. Two-way ANOVA analysis of c-Fos expression in the SCN revealed a significantly different reaction to treatment (*ad libitum*, fasting, and glucose) between SHAM en RC-cut animals (*F*_(2,25)_ = 3.489, *p* = 0.0461; Fig. 5). SHAM animals drinking 5 ml of a 3% glucose solution following 48 h of fasting showed a strong decrease in c-Fos expression in the SCN (*p* < 0.01) as compared with *ad libitum* conditions. Conversely, RC-cut animals showed no significant change in c-Fos expression in the SCN (*p* > 0.05; Fig. 5). Also, merely fasting, known to result in a moderate decrease of c-Fos in the ventral SCN (Saderi et al., 2013) did not result in any change in the RC-cut animals. Furthermore, a comparison of SHAM and RC-cut animals showed a significantly elevated SCN c-Fos expression in RC-cut animals in all conditions as compared with SHAM animals.

Also in the ARC, c-Fos expression in reaction to *ad libitum*, fasting, or glucose was significantly different in RC-cut animals as compared with SHAM animals (*F*_(2,25)_ = 15.59, *p* < 0.0001). In reaction to the glucose stimulus, the ARC in SHAM animals showed a significant increase in c-Fos (*p* < 0.01) as compared with *ad libitum* conditions, while RC-cut animals, again, demonstrated no significant difference between conditions (*p* = 0.0775). Thus, SHAM animals showed a decrease in SCN c-Fos activity that coincided with an increase of c-Fos in the ARC. This reciprocal influence is clearly lost in RC-cut animals, which showed no significant change in c-Fos expression in the SCN or ARC between different conditions.

### Deafferentation of SCN-ARC neuronal connections does not damage their efferents to other brain areas

**Figure 6.**
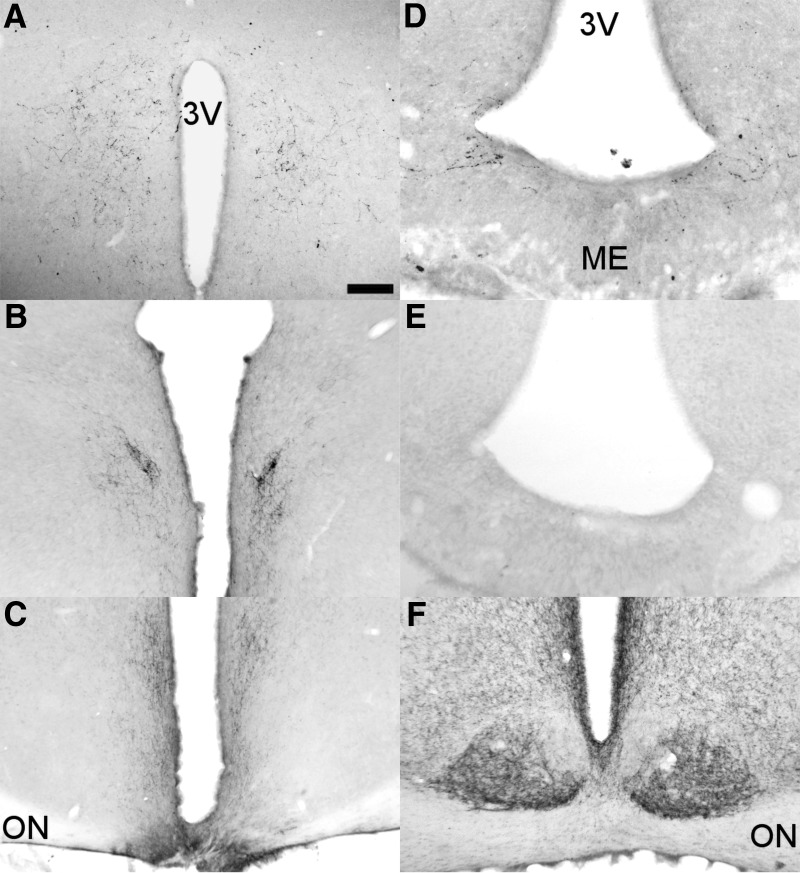
RC cuts do not affect main SCN output other than the ARC. Clear VIP innervation of the DMH (***A***) and PVN (***B***) in RC-cut animals. **C**, Also VIP staining to the SPZ and retrochiasmatic area dorsal to the knife cut is still intact in RC-cut animals. ***D***, Staining of VIP afferents in the ARC, clearly visible in SHAM animals, disappear following RC cut (***E***). ***F***, Likewise, NPY SCN staining, shown to be predominantly IGL derived, is not damaged by RC cuts. Pictures ***A–C***, ***E***, ***F*** are from RC animals, only ***D*** shows staining in an intact animal. Scale bar, 130 μm (***A*** and ***F***), 175 μm (***B***), 250 μm (***C***), 70 μm (***D*** and ***E***).

Since for most of our observations we used knife cuts in the retrochiasmatic area, we investigated whether this might have also severed other connections between the SCN and its target areas, other than the ARC. Also, we established whether knife cuts removed ARC output to efferent brain areas, especially dorsal and rostral of the cut. Immunohistochemical staining for VIP, recognized as one of the major output systems of the SCN (Takahashi, 1989), revealed that, except for its projections to the ARC, VIP innervation to the SPZ, PVN, and DMH remained unaffected (Fig. 6*A*--*C*). VIP innervation of the ARC and the area between the median eminence and ARC, present in SHAMs, disappeared in RC-cut animals (Fig. 6*D*,*E*). No statitical analysis was performed on the extent of loss or preservation of innervation since results were binary. Also NPY staining in the SCN reflecting input from the IGL (Fig. 6*F*) was not affected by RC cuts. Through unilateral ARC CtB injections in animals with ipsilateral RC cuts, we confirmed the loss of SCN connections with the ARC as compared with controls (data not shown).

**Figure 7.**
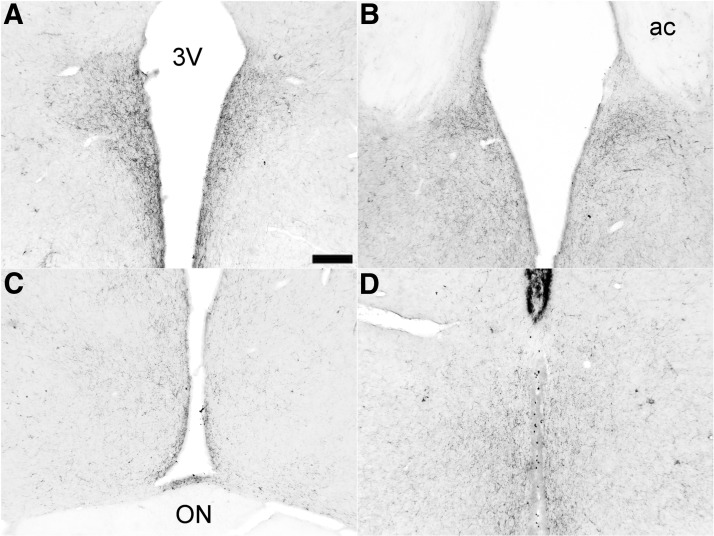
ARC-specific AgRP staining shows intact innervation of (***A***) the PVN, (***B***) MnPO, (***C***) AVPV, and (***D***) DMH, not affected by the knife cuts. Scale bar, 175 μm (***A–C***) and 215 μm (***D***). All photomicrographs are from RC-cut animals. 3V, third ventricle; ON, optic nerve.

**Figure 8.**
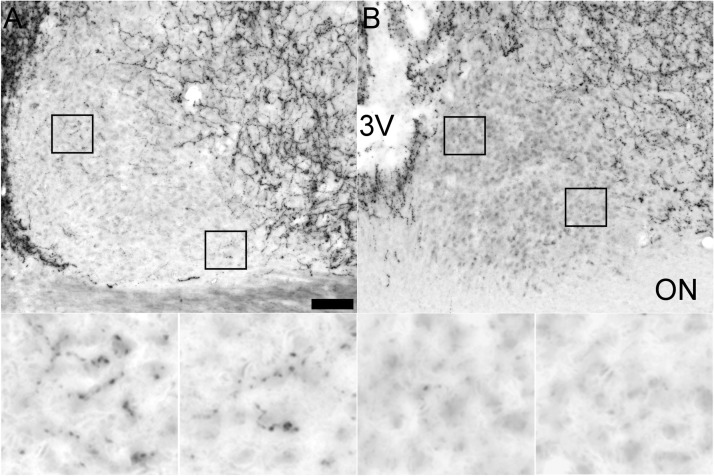
RC cuts strongly temper ARC innervation of the SCN as illustrated by diminished of ARC-specific AgRP staining. Representative photomicrographs of AgRP staining showing intact innervation of (***A***) the SCN in a SHAM animal. ***B***, SCN of an RC-cut animal with strongly reduced AgRP innervation. Bottom pictures are magnifications of outlined boxes in ***A*** and ***B*** showing clear fibers in the SCN of control animals not seen in RC-cut animals. Scale bar, 70 μm (***A*** and ***B***), and 15 μm (the magnifications below). 3V, third ventricle; ON, optic nerve.

Analysis of ARC-specific output by means of AgRP staining showed a normal distribution of AgRP in RC-cut animals in main ARC target areas, i.e., the POA, PVN, VMH, and DMH, suggesting normal ARC output unimpaired by the retrochiasmatic knife cut (Fig. 7). Assessing the completeness of SCN denervation from ARC efferents, AgRP staining showed a clear attenuation of SCN innervation in RC-cut animals compared with SHAMs (Fig. 8). Together with the unilateral SCN lesion and contralateral knife cut, these anatomic analyses demonstrate that reciprocal connections between the SCN and ARC are affected by our microcuts, but other SCN and ARC efferents remain intact.

## Discussion

Most physiologic processes have well-defined circadian rhythms. The present study challenges the still prevalent view that these rhythms are driven solely by the SCN. We demonstrate that generation of specific physiologic and behavioral circadian rhythms greatly depend on functional interaction between the SCN and ARC. RC cuts, effectively eliminating reciprocal communication between the SCN and ARC, resulted in a complete loss of rhythm in corticosterone secretion, whereas during DD conditions also locomotor activity and body temperature rhythms were completely lost. These findings are in agreement with prior studies demonstrating a role for the mediobasal hypothalamus in facilitating circadian rhythmicity (Gerkema et al., 1990) and consistent with observed loss of rhythm in adrenal corticosterone levels following knife cuts posterior to the SCN (Moore and Eichler, 1972). In the present study, we show that the reciprocal connectivity between SCN and ARC (Saeb-Parsy et al., 2000; Yi et al., 2006) is essential to synchronize hormonal and behavioral rhythms.

### Interaction between SCN and ARC is essential for generating rhythms

The striking observation that corticosterone rhythmicity was lost in RC-cut animals during LD conditions suggests a crucial role for the ARC in generating this rhythm jointly with the SCN. A recent observation that glucocorticoid receptors in the ARC are essential for the circadian variation in the negative feedback of circulating corticosterone on its own release (Leon-Mercado et al., 2017) supports this observation. Locomotor activity, rhythmic in LD, was completely arrhythmic in DD. One could argue that considering locomotor activity is rhythmic in LD conditions, for maintaining this rhythm in DD interaction between SCN and ARC is essential. On the other hand, there is the suggestion that the observed behavioral phenotype in LD is not truly rhythmic but rather shows diurnal variance due to the “masking” effect of light (Redlin and Mrosovsky, 1999). In view of the effect of locomotor activity on temperature, the rhythmic locomotor activity in LD in RC-cut animals may also be responsible for the observed rhythmic temperature in LD, while lost in DD. The present results show that for maintaining rhythmicity in DD, interaction between SCN and ARC is essential.

The SCN receives numerous types of nonphotic feedback signals capable of altering the phase of the clock. Activity can synchronize and phase-shift the SCN (Yamazaki et al., 1998; Schaap and Meijer, 2001), SCN clock genes can be synchronized to food in LL conditions (Lamont et al., 2005), while extra-SCN areas can influence the phase of SCN clock gene, *Per1* (Vansteensel et al., 2003). Considering this, we hypothesized that severed SCN-ARC connections resulting in diminished nonphotic, metabolic feedback from the ARC to SCN (Yi et al., 2006) as well as aberrant rhythms in behavior, temperature, and corticosterone could lead to suppression of SCN rhythmicity. Yet, SCN rhythmicity measured by Per1 expression remained unaltered in RC-cut animals. This observation suggests that, in the short term, the SCN is able to maintain its own autonomous rhythm in clock gene expression and that to drive physiologic functions, it depends on its interaction with the ARC. The observation that SCN-lesioned animals receiving SCN transplants from donor animals regain circadian rhythmicity, suggests that they do not require ARC interconnectivity. However, it has been shown that diffusible substances from the transplanted SCN into the third ventricle also restore locomotor activity rhythms (Silver et al., 1996). Since the ARC, as a circumventricular organ, takes up substances from the third ventricle, it could be an effective target for SCN-derived diffusible substances.

Further assessing hypothalamic output through peripheral clock gene rhythmicity, we found liver *Per1* expression of experimental animals to be similar to SHAM animals. However, no rhythm could be detected in liver *Bmal1* and *Cry1* clock gene expression. This anomaly could be explained by the fact that while there is no rhythm in corticosterone, activity, temperature, and possibly food intake (Li et al., 2012), the persistent rhythm in melatonin could have its effect on liver *Per 1* expression (Houdek et al., 2016).

### Circadian rhythms depend on synchronous SCN and ARC output

A recent study has demonstrated that SCN driven activity of ARC α-MSH neurons is essential for diurnal temperature control (Guzman-Ruiz et al., 2015). Moreover the study demonstrated that synchronized release of SCN-derived vasopressin, and ARC-derived α-MSH in the medial preoptic area is essential for the physiologic dawn temperature decrease in rats. According to our present observation of an altered ARC PER1 rhythm in RC-cut animals, disclosing a desynchronous state of the ARC from the SCN, should indeed lead to a loss in temperature rhythm. The necessity of ARC and SCN synchrony is also consistent with data showing the ARC is essential for the regulation of activity and feeding. Lesions targeted at specific neuronal populations in the ARC resulted in deteriorated activity, temperature, and feeding rhythms (Coppari et al., 2005; Li et al., 2012). Our results indicate, it is not the ARC lesions per se, but an altered phase relationship between the SCN and ARC leading to desynchronized SCN-ARC output with a deleterious effect on physiologic function. As such, the observation that RC-cut animals maintain a normal melatonin rhythm in LD and DD conditions affirms that melatonin is solely dependent on SCN activity (Pévet, 2014), while rhythms in locomotor activity, temperature, and corticosterone levels are also dependent on regulation by the ARC and thus arguably affected by changes in metabolism.

### Technical considerations

Making knife cuts inevitably causes collateral damage, and while keeping this to a minimum (as shown in Figures 1 and 6), we cannot exclude consequential damage to neurons and pathways to and from other areas, perchance contributing to observed rhythmic aberrations. Retrochiasmatic lesions for example, have been shown to reduce pineal melatonin content (Ribeiro Barbosa et al., 1999); however, in the present study, the rhythm in melatonin secretion was not altered and animals with knife cuts placed out of midline, still transecting the retrochiasmatic area, did not express any change in rhythmicity. The SPZ, located above the area of the knife cut, is a target of neural projections from the SCN (Watts et al., 1987) and functions as a possible relay for the SCN (Cipolla-Neto et al., 1988), suggesting that a loss of SPZ input could be responsible for observed change in rhythmicity. This, however, seems unlikely for two reasons; first, we show the SCN projections to the PVN, SPZ and DMH are still intact (Fig. 6). Second, other studies demonstrated that horizontal knife cuts eliminating SCN input to the SPZ reduced the LH surge in female rats but did not reduce the rhythmicity of locomotor activity, nor in constant conditions (Watts et al., 1989), indicating that the SCN projections to the SPZ are not essential for rhythm in locomotor activity.

Realizing that knife cuts could also sever unforeseen projections we investigated SCN and ARC projections to and from other hypothalamic areas. The presence of NPY input from the IGL to the SCN was unaffected and also ARC and SCN output to other areas was not notably altered. Still the possible disruption of other SCN or ARC afferents could be considered a limitation of the knife cut technique, restricting a conclusion about the SCN-ARC interaction. Thus, to reduce this possibility we performed a unilateral SCN lesion with a contralateral knife cut. Unilateral SCN lesions alone allow an animal to maintain its circadian rhythm (Gerkema et al., 1990; Guzmán-Ruiz et al., 2014). Hence, to ensure our observations were the result of severed interaction between the SCN and ARC and not due to damage of the retrochiasmatic area or other hypothalamic projections to the SCN and ARC, we combined unilateral SCN lesions with a contralateral knife cut, leaving the unilateral SCN, unilateral retrochiasmatic area and complete ARC intact. With this experiment, again preventing reciprocal communication between SCN and ARC, we demonstrate equal aberrations of physiologic rhythms as seen following a complete RC cut. In combination with demonstrated intact SCN (VIP) and ARC (AgRP) efferents to other hypothalamic target areas, we show it is the eliminated SCN-ARC communication responsible for observed loss of rhythmicity in physiologic outputs and not damage to other hypothalamic nuclei or circuits. Since we investigated the nature of SCN-ARC interaction consisting of various, unknown, neuronal populations, a Cre recombinase animal study would not be appropriate for this specific purpose. However, it could prove applicable in future research, investigating the character of involved individual neuronal populations and their function in SCN-ARC interaction.

### The SCN and ARC depend on interaction to modulate their activity in response to metabolic cues

The rhythmic SCN *Per1* expression together with an intact melatonin rhythm demonstrates preserved SCN rhythmicity. However, RC-cut animals responded to metabolic cues not only with an altered neuronal activation of the ARC but also of the SCN, illustrating that for proper function both the ARC and SCN depend on their interconnectivity through the SCN-ARC axis. This is consistent with observations that altered metabolic conditions of an animal change the activity of the ARC and that of the SCN simultaneously (Yi et al., 2008; Saderi et al., 2013). Moreover, recent findings show that the SCN readily modulates glucose sensing in the ARC in a time dependent manner (Chao et al., 2016) and light not only directly changes the neuronal activity of the SCN but also that of the ARC (Guzmán-Ruiz et al., 2014) further confirming the significance of the SCN-ARC axis.

## Conclusion

The present results provide evidence that SCN-ARC interaction serves to synchronize SCN and ARC output, essential for organizing physiologic functions. This confirms the idea, also previously suggested (Webb et al., 2009; Hu et al., 2012; Buijs et al., 2016), that the SCN functions inside a larger circadian network of tightly linked oscillatory feedback circuits whose integral function is essential for regulating physiologic and behavioral functions. Long-term desynchronization within this circadian network due to changes in dietary habits, chronic jetlag, or shift work is known to contribute to pathology associated with “modern lifestyle,” such as hypertension, obesity, diabetes, and cancer (Scheer et al., 2010; Leproult et al., 2014; Kettner et al., 2016). We therefore propose that ill-timed food intake and altered metabolic signals are able to alter normal ARC activity patterns, as such changing its synchronization with the SCN and as a consequence disrupting associated behavioral and hormonal patterns. Thus, faulty network connections or erroneous feedback may reshape the circadian system to a new equilibrium, leading to physiologic impairment and pathology.

## Synthesis

Reviewing Editor: Rae Silver, Columbia University Decisions are customarily a result of the Reviewing Editor and the peer reviewers coming together and discussing their recommendations until a consensus is reached. When revisions are invited, a fact-based synthesis statement explaining their decision and outlining what is needed to prepare a revision will be listed below. The following reviewer(s) agreed to reveal their identity: Tamas Horvath, Quentin Pittman

In this study, reciprocal connections between the SCN and the Arcuate ( ARC) were severed with a knife cut. By examining a variety of circadian rhythms in complete darkness (DD), the authors could examine the role of the SCN-ARC reciprocal connection in maintaining free running rhythms. The data show that locomotor activity, temperature and corticosterone rhythms, but not melatonin rhythms are abolished, as is synchronicity of the ARC by these SCN-Arc cuts. In addition, two transcription factors in the liver, but curiously not Per1 were also affected.

As expected, circadian periodicity of the SCN ( Per1) was maintained. In addition the ability of a glucose load to alter reciprocal cFOS in the SCN and ARC was disrupted. Special care was taken to deal with the possibility of nonspecific retrochiasmatic damage as well as disruption of a number of other SCN outputs- thus ruling these out.

Quality of the immunohistochemistry is good and the evidence for the lack of effect of the lesions on other outputs is fairly compelling. Significance statement is good.

Comments:

1. The take home message appears to be that the SCN does not operate in isolation in sustaining rhythmic locomotor and some other rhythms, but is influenced by the ARC - presumably via metabolic inputs. That said, there is some confusion of the wording of the conclusions. The idea of SCN-ARC reciprocity being critical seems inappropriate given that SCN rhythmicity was maintained. Rather, it appears that reciprocity may be necessary for rhythmicity in some responses that are regulated by SCN output s to the ARC. This important point could be elaborated /clarifies.

2. A major aspect of the work is the SCN lesion, either unilateral or bilateral, the transection of the SCN/ARC connection and the combination of the unilateral SCN lesion with the ARC transection. These are tricky manipulations. It is unclear, as written, whether all animals in which these manipulations were performed were successful, or whether a larger number of animals were performed and only after histological verification, they were included in the results..In fact, the reporting of the numbers of animals that were attempted and used, and ( if) why some were dropped measured must be reported for a number of the manipulations.

3. The order in which the figures are dealt with the text is disruptive to the reader. Consider moving the last paragraph of the results to the text dealing with Fig 1 in the methods.

4. It seems that reference in the text to Fig 6A,B,C,F are missing.

4. Why is there a significant difference in the melatonin groups at ZT18?

5. ZT and CT should be defined and explained for the non-specialist reader. Responses in LD should be described as diurnal rather than circadian.

6. While the immune figures are clear, it would be useful to know how many animals were represented for the various immunos; was there any attempt at quantification? ( eg Fig8)?

7. Line 181: What is an overt animal? Overt animals were used for 182 immunohistochemistry and in situ analysis.

8. Line 166 176 201 and elsewhere. Please indicate the LD cycle in which animals were held, for each of the different measures. (e.g.“Chi-squared periodogram analysis of locomotor activity in 316 light-dark (LD, 7 days, n=7), showed a significant circadian rhythm in both SHAM 317 and RC-cut animals”

9. Correction needed: Tuckey's multi-comparison post-hoc test : should be Tukey

In reporting the ANOVAs please indicate what factors were analyzed for each analysis, as this is often not evident.

10. Line 201ff: Please amplify for the nonspecialist the explanation of how this protocol tests the “relevance” of the connection.
